# Evaluation of the Use of Shared Decision Making in Breast Cancer: International Survey

**DOI:** 10.3390/ijerph18042128

**Published:** 2021-02-22

**Authors:** Marta Maes-Carballo, Manuel Martín-Díaz, Luciano Mignini, Khalid Saeed Khan, Rubén Trigueros, Aurora Bueno-Cavanillas

**Affiliations:** 1 Unidad de Patología Mamaria del Servicio de Cirugía General, Complexo Universitario Hospitalario de Ourense, 32005 Ourense, Spain; marta.maes.md@gmail.com; 2 Department of Preventive Medicine and Public Health, University of Granada, 18014 Granada, Spain; profkkhan@gmail.com (K.S.K.); abueno@ugr.es (A.B.-C.); 3 Hospital Básico Santa Ana de Motril, 18600 Granada, Spain; vistamar7@gmail.com; 4 Unidad de Mastología del Grupo Oroño, 2000 Rosario, Argentina; lmignini@gmail.com; 5 CIBER of Epidimiology and Public Health (CIBERESP), 28029 Madrid, Spain; 6 Department of Language and Education, University of Antonio de Nebrija, 28015 Madrid, Spain; 7 Instituto de Investigación Biosanitaria (IBS), 18012 Granada, Spain

**Keywords:** shared decision making, breast cancer, use of shared decision making, survey, longitudinal study

## Abstract

Objectives: To assess shared decision-making (SDM) knowledge, attitude and application among health professionals involved in breast cancer (BC) treatment. Materials and Methods: A cross-sectional study based on an online questionnaire, sent by several professional societies to health professionals involved in BC management. There were 26 questions which combined demographic and professional data with some items measured on a Likert-type scale. Results: The participation (459/541; 84.84%) and completion (443/459; 96.51%) rates were high. Participants strongly agreed or agreed in 69.57% (16/23) of their responses. The majority stated that they knew of SDM (mean 4.43 (4.36–4.55)) and were in favour of its implementation (mean 4.58 (4.51–4.64)). They highlighted that SDM practice was not adequate due to lack of resources (3.46 (3.37–3.55)) and agreed on policies that improved its implementation (3.96 (3.88–4.04)). The main advantage of SDM for participants was patient satisfaction (38%), and the main disadvantage was the patients’ paucity of knowledge to understand their disease (24%). The main obstacle indicated was the lack of time and resources (40%). Conclusions: New policies must be designed for adequate training of professionals in integrating SDM in clinical practice, preparing them to use SDM with adequate resources and time provided.

## 1. Introduction

Breast cancer (BC) is the leading cause of death in women [1]. Improvements in diagnosis, the greater efficacy of neoadjuvant therapies and the development of new oncoplastic techniques and oncological management have reduced the aggressiveness of surgical treatments and improved the aesthetic and functional results [2]. As BC treatment is now more complex, each case’s ideal approach requires a high degree of individualization, scientific-technical updating, multidisciplinary coordination, and continuous review of results [3].

The ideal strategic plan for a BC patient will be the one that best meets their needs and expectations. Its design should be based on an accurate diagnosis of their disease and the patient’s circumstances, preferences, and values [2,3]. So, shared decision making (SDM), “*an approach in which physicians and patients share the best available evidence when faced with the task of making decisions and where patients are supported in considering options, to achieve decisions following their preferences and values*” [4], is vitally important in BC. Its diagnosis and treatment requires multiple high-risk decisions made in a limited time period and, often, with incomplete evidence, raising the need for more significant patient support during their decision-making process [4].

SDM is a universally supported concept [5,6,7] linked to care quality [8,9]. It increases patient satisfaction and their perception of risk [10]. It is a legal obligation in large parts of developed countries [11,12,13,14] and reduces malpractice claims [15,16]. However, its actual implementation remains low [17,18]. It is poorly reflected in clinical practice guidelines and consensus [19] and obstacles to its implementation persist [20,21]. Its main objective is to respect patients’ autonomy without detriment to their benefit, providing care under their values and preferences. This requires the development of multidisciplinary teams with a high scientific-technical level, excellent coordination, communication with the patient, and permanent review of the results within the framework of a continuous improvement program.

The aim of this work is to assess the level of interest, knowledge and attitude towards SDM, as well as the perception of the degree of application of SDM by health professionals involved in the management of BC (including the entire process screening, diagnosis, treatment and follow-up).

## 2. Methods

The “Checklist for Reporting the Results of Internet E-Surveys” (CHERRIES) was used for this study, which allows a quality description of the research results from surveys of web environments [22,23]. CHERRIES, used for ensuring complete descriptions of e-survey methodology, is designed to improve the quality of reports [22]. A cross-sectional observational study on a convenience sample of BC specialist was conducted.

### 2.1. Measurement

A questionnaire was designed by a group of three SDM experts and breast cancer specialist (MMC, MD, LM) with a comprehensive theoretical and practical experience about this deliberative [24,25]. A literature review about SDM was done to elaborate and design a questionnaire to be self-completed online (Appendix A), which included brief information on the study’s scope and objectives and a warning to those members of several of these societies not to answer it in duplicate. The survey was constructed in Spanish (Spanish and Argentine variations). Both variants were reviewed by native authors (MMC for Spanish from Spain and LM for Spanish from Argentina). No identifying data were collected. The variables of interest were measured on a Likert-type scale [26,27] with 5 responses, 1 being “strongly disagree” and 5 “strongly agree”. The degree of knowledge about SDM (questions 1–5), the opinion about SDM (questions 6–12), the awareness and attitude about SDM (questions 13–15) and the degree of current and future application of SDM (questions 16–23) were investigated. Finally, three open-ended questions were included, referring to the perceived advantages, disadvantages and obstacles to its implementation. An arbitrator (ABC) has reviewed this prototype questionnaire and suggested modifications. Prior to disseminating the questionnaire, a pilot test was carried out on a sample of 15 specialists contacted directly to assess the questions’ understanding and relevance. Some modifications for improving understanding of the survey have been done.

We could not estimate the response or participation rate. The completion rate was calculated from those who opened the online link. The real participation rate was impossible due to open distribution dissemination [28,29].

### 2.2. Period and Scope of the Study

The information was collected during the months of June, July, August and September 2020 in two countries: Spain and Argentina. The reference population was BC treatment specialists, members of scientific societies related to this process (BC screening, diagnosis, treatment and follow-up): Asociación Española de Cirujanos (AEC), Sociedad Española de Senología y Patología Mamaria (SESPM), Sociedad Argentina de Mastología (SAM), Sociedad Argentina de Cirugía Plástica, Estética y Reparadora (SACPER), Asociación de Oncología de Rosario (AOR) y Asociación de Mastología de Rosario (AMAR). The sample was made up of the members of these societies who received and answered the online survey. Surveys that did not answer at least 25% of the items surveyed were excluded.

### 2.3. Data Collection

The participating scientific societies sent the survey by e-mail to the partners’ list, included a link on their websites and the possibility of sharing this link with other colleagues. Two reminders were sent after the initial invitation; all constructed by the team researcher. The response was entirely voluntary and without incentive. It was administered through Google Forms [30], an online survey platform, from 1 June to 31 October 2020. There was no obligation to answer all the questions, and backtracking was allowed to answer previous questions. There was no random assignment of questions and answers. No data identifying the participants were stored. No minimum completion time was specified a priori. Partially completed surveys were accepted, provided that at least 25% of the questions were answered, and a manual review was conducted to verify abnormal response patterns.

### 2.4. Data Analysis

The distribution of responses and the average values of each question of the survey were studied, stratifying by sex, age, professional seniority, speciality, type of hospital (public or private) and service (with or without breast unit), and the number of patients attended annually, by the professional and by the hospital. The results were compared using Chi-square test to compare proportions (Table 1), a mean comparison test for independent groups (Student T-test) to compare across two categories of variables (Table 2) or analysis of the variance of one route (ANOVA with Bonferroni correction) for variables with more than two categories. Statistical significance was set at *p* < 0.05. All analyses were performed with the Stata 15.0 statistical package (StataCorp LLC, College Station, TX, USA).

## 3. Results

A total of 541 doctors viewed the survey, and of these, 459 (84.84%) provided demographic information and answered at least 25% of the questions and one question based on content (participation rate). The majority of participants (443/459; 96.51%) completed all questions (completion rate). There were only 5% of unanswered questions, which was not significant. No pattern to the unanswered questions was found.

### 3.1. Participants

Table 1 summarised the socio-demographic and professional characteristics of the participants and compared then between countries. There was a similar representation of both sexes, mostly under 50 years old, with various specialities distribution. Most participants belonged to a breast unit (71.42%; *p* = 0.001), but only one third worked in hospitals with more than 200 cases per year (31.06%; *p* = 0.001). When comparing between Argentina and Spain, differences in age (younger professionals in Argentina) and the speciality stand out. A total of 51.26% of Argentine professionals were classified as mastologists, a speciality that does not exist in Spain and which is replaced by 56.25% of general surgeons (*p* = 0.001). It was more frequent in Spain than in Argentina to belong to a breast unit (88.33% vs. 39.70%; *p* = 0.001) and work in a public hospital (76.79% vs. 39.50; *p* = 0.001).

### 3.2. Global Analysis of the Survey and Comparison between Countries

Table 2 presents the results of the questionnaire. The majority responses were in all cases values 4 “agree” and 5 “strongly agree”, except for question 22. The first five questions, about the degree of knowledge of the SDM, obtained a high concordance. Only in the first case, there was a slightly higher score in the Argentine participants (4.51 vs. 4.33), but still statistically significant (*p* = 0.027). The opinion about SDM questions (questions 6-12) revealed a very positive attitude about SDM, which was higher for Argentinean surgeons in terms of the usefulness of SDM in the relationship with patients (question 6, 4.79 vs. 4.33; *p* = 0.001), also obtaining a higher score in the obligation to explain to patients (question 9, 4.77 vs. 4.67; *p* = 0.036). The Spanish were more willing to help patients understand the information (question 10, 4.73 vs. 4.35; *p* = 0.001) and ask about their expectations (question 11, 4.46 vs. 4.19; *p* = 0.001).

Concerning the questions that measured attitude and awareness about SDM (questions 13–15), question 13, on providing sufficient time, also obtained a high level of agreement, greater in the Spanish practitioners (4.25 vs. 4.54; *p* = 0.001). All these results are presented in Table 2. Question 14, on the joint choice of treatment, also got an enormous agreement but without significant differences between countries (*p* = 0.135). However, when it comes to monitoring the process, question 15, the degree of agreement decreased, particularly in Argentina (3.80 vs. 3.65; *p* = 0.001). Regarding the degree of current and future application of SDM (questions 16–23), the survey obtained the lowest values. Question 17, on the existence of a specific consultation (3.41 vs. 3.26; *p* = 0.179), and questions 18 (3.63 vs. 3.24; *p* = 0.001) and 19 (3.61 vs. 3.29; *p* = 0.001), on the availability of the necessary time and resources respectively, got lower results in Spain. There was high agreement on the need for more training (question 21), significantly higher in Argentina (4.41 vs. 4.25; *p* = 0.023), and on the future growing application (question 23). There was low agreement on Spain’s public and private assistance than Argentina (1.65 vs. 2.49; *p* = 0.001).

When the responses were stratified by sex, the highest score obtained by women for questions 9 (4.80 vs. 4.64; *p* = 0.004), 10 (4.61 vs. 4.44; *p* = 0.007) and 11 (4.40 vs. 4.23; *p* = 0.009) stood out, revealing a more empathetic attitude on the part of the women, who in turn are more aware of the need for SDM as a quality tool, question 2 (*p* = 0.003). In contrast, men were more likely to consider the doctor the most appropriate person to decide, question 12 (*p* = 0.033). Regarding age, significant differences in favour of younger professionals (doctors more youthful than 50 years old) were observed for questions 6–9, related to attitude, and for that referring to a future application, question 23 (4.41 vs. 4.24; *p* = 0.041).

When analysing the answers by speciality, the highest degree of agreement of the specialists in mastology concerning questions 1 (knowledge of the fundamentals of SDM), 6 (SDM as a basic element of the relationship with the patients), 8 (obligation to explain) and 12 (the patient believes that the doctor should choose the treatment) stood out. Argentinian had more time (question 18) and were more predisposed to recognise differences between public and private care (question 22). Plastic surgeons stood out for the greater agreement regarding the usefulness of SDM when there were several alternatives (question 3) and the need to explain the different treatment options (question 7), their advantages and disadvantages (question 9), and the need for further training (question 21). Finally, the general surgeons claimed the need to help patients understand the information (question 10) and the necessity of time to do so (question 13). Concerning the existence of a Breast Unit, there were few significant differences. However, when there was one, more emphasis was placed on incorporating the patient into the follow-up process (question 15), and the greater experience was highlighted (question 16). On the other hand, when not working in a breast unit, the results were higher for question 6 (SDM as a basic element of the relationship with patients), 8 (obligation to explain) and the need for the joint choice of treatment with patients (question 14), but they also agreed that patients generally consider that it is the doctor who should decide (question 12).

### 3.3. Advantages, Disadvantages and Main Obstacles to the Implementation of the SDM

**Figure 1:**
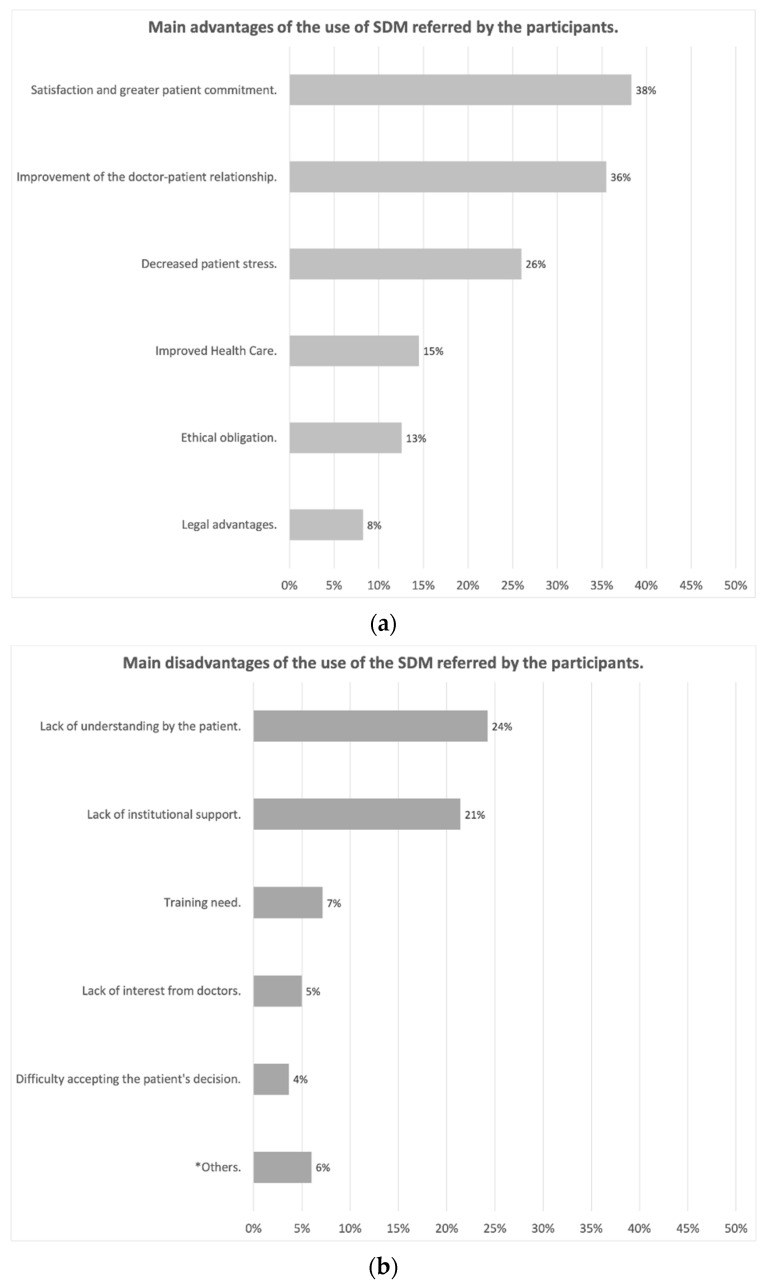
(**a**) Main advantages of the use of the SDM referred by the participants; (**b**) Main disad-vantages of the use of the SDM referred by the participants (* Others: lack of universality, delay in the patient’s decision and difficult applicability).

**Figure 2:**
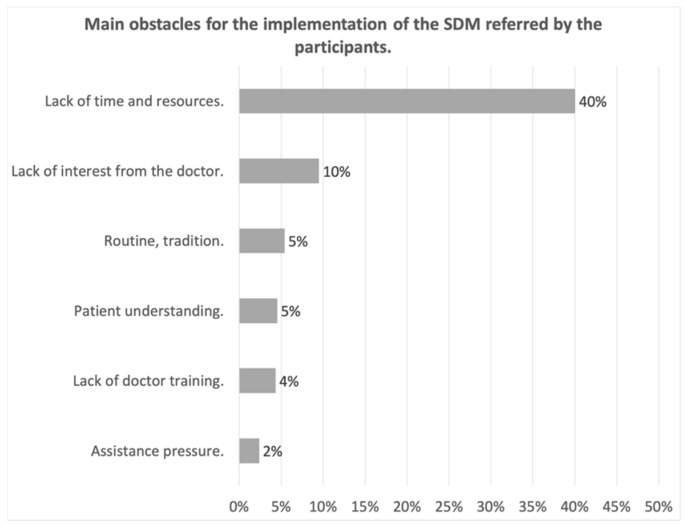
Main Obstacles for the implementations of the SDM.

Figure 1a and b shows the main advantages and disadvantages of SDM, as reported by participants. The main advantages highlighted were patient satisfaction and greater commitment to treatment (38%), improvement in the doctor-patient relationship, thus increasing confidence in the doctor (36%) and reduction in patient stress by helping them to understand their illness (26%). The main drawback was the lack of patient literacy (24%) followed by the lack of institutional support, lack of means, and time in consultation to implement it (21%). Concerning the obstacles, Figure 2, widely highlighted the lack of time and resources or materials (a proper SDM consultation available, training courses for practitioners, …) for the implementation of SDM, pointed out by 40% of the respondents.

## 4. Discussion

Most of the professionals who answered the survey had a broad knowledge and a favourable opinion about SDM. Spanish speaking practitioners were keener to help the patient understand the information process and ask about patient expectations. More Argentineans thought about SDM as an essential element in BC management and an obligation to pursue. Regarding the awareness-raising and attitude about SDM, participants, mainly Spanish, agreed on the necessity of providing enough time to practice SDM and on the joint choice of treatment. Concerning the current and future application of SDM, there was high agreement on the need for more training. The least agreement was observed for the necessity to agree with patients on the process’s follow-up and the current and future implementation. This was mainly in the availability of specific consultations or time and resources for SDM in the participant service. On the other hand, participants highlighted patient satisfaction and a more significant commitment to treatment as the main advantage of SDM and the lack of patient preparation to understand their illness as the main drawback. They pointed out the lack of time and resources as the main obstacle.

### 4.1. Strengths and Limitations

The design and presentation of the study have followed the CHERRIES publication guideline [22,23], so necessary measures have been taken to maintain the quality required in this type of research. The results were underpinned by the inclusion of a significant number of participants, 459, all from different specialities and periods of professional careers in Europe and Latin America, with very different health systems [31].

The lack of established psychometrics of the survey could be considered a limitation. However, this psychometric validation aimed typically to adapt and validate an instrument to measure elements of frequently ambiguous context. In our study, knowledge and attitudes were measured without quantifying or integrating the responses into a complex index.

The main limitation results from the participants’ selection bias implicit in online surveys, which possibly leads to responses in favour of SDM. Social desirability bias was inherent to this kind of survey. It could have led professionals to answer based on social expectations rather than their real attitudes towards SDM [32]. Anonymity and confidentiality of the answers were used to reduce it [33]. Therefore, the possible existence of a selection and social-desirability bias further reinforces the results obtained: even among those professionals most likely to use SDM, there is a lack of use, and in particular of time and resources.

On the other hand, sending the survey by open distribution made it impossible to estimate the real response rate [28,29]. E-mail distribution of surveys has a lower response rates than other distribution routes such as telephone surveys [29]. Fortunately, previous reviews identified smaller-than-anticipated differences between physician respondents and non-respondents and between early and late responders [34,35,36], suggesting low nonresponse bias rates [28]. The completion rate was high, suggesting recognition of the importance of this issue to quality health practice today.

The questionnaire was validated by a pilot test sent to fifteen BC specialist. We were using questions to explore concepts, believes and attitudes. No other tools were found useful to measure these aspects, so we did not have a gold-standard to validate how accurately the selected questions assess every domain (knowledge, opinion, awareness-raising and attitude about SDM, and current or future application of it). Lack of answer variability is problematic in telemedicine surveys because of its harmful effects in responses sensitivity and reliability. This ceiling effect resulted from high satisfaction ratings. Although one presumed solution would be to create a rating scale with more significant discrimination of responses in the continuum scale [37], some studies have found the number of rating points unrelated to cross-sectional reliability [38,39]. There was not enough evidence to support this statement [37]. We have created a 5-point Likert scale that has been demonstrated assurance before [26,27].

Regarding comparisons referring to 23 items as a dependent variable, we could suppose that part of the differences detected might be due only to chance. This was an additional limitation, mainly when the effect of age, sex, size and setting of the hospital and the participant’s speciality has been analyzed for each item. Determined patterns have not been appreciated, and the results were interpreted with great caution.

Regarding participants’ characteristics, most of the participants did not belong to breast units. This was possibly due to the high requirements necessary to constitute a breast unit [40], which means that there were not too many breast units in hospitals in absolute numbers. A more decisive data were the number of patients treated by each participating physician. A total of 46.97% of the participants treated more than 100 patients per year, a significant number of cases in individual terms and allowed consistency to the findings found in this study.

It has also been shown that participants under 50 years old were opener to SDM. However, it might probably influence that doctors under 50 years of age were more familiar with our survey’s distribution networks. However, a more precise analysis could observe that most participants were under 50 years of age because they were the vast majority of active workers in BC today. In the majority of the countries, the retirement age is contemplated from 65 years. Moreover, apart from the fact that this older population would presumably be less interested in updating their knowledge, it was also less interesting for our study since they did not represent active BC management work.

### 4.2. Implications

To our knowledge, this study was the first international survey of BC specialists on the understanding, attitude and application of SDM. This was surprising as SDM is an essential component of quality health care [8,9] and a legal obligation in most developed countries [11,12,13]. The practice of SDM in cancer care has been proposed as a crucial element to change a system’s course in crisis towards excellence and sustainability [4]. Its implementation in BC care constitutes a very demanding path, which implies the creation of multidisciplinary teams with a high scientific-technical level, excellent coordination, continuity of care and communication with the patient, and a persistent review of the results of a continuous improvement program. Although there are no previous studies of the environmental impact that SDM could cause, it would be logical to think that increasing the efficiency and quality of BC management would reduce the use of resources. This would ultimately be one more foothold to impulse the use of SDM. More studies should be done to support this statement.

As no similar work about SDM in practitioners has been done before, comparisons between researches were impossible to obtain. Therefore, this highlights the importance of this study because the findings were significant in themselves. The study’s basis and design were very innovative. Previous surveys done about SDM were about patients’ perception and experience [41,42,43]. All these studies reported a low application of SDM. Moreover, a similar study was done in medical students with similar knowledge results [44]. Still, as they were participants in training and not practitioners, the study was limited since they could not put SDM into practice.

The results refer exclusively to BC, a disease that highlights the importance of SDM in cancer care management. In BC, the different alternatives that exist require an exchange of information between doctor and patient and the inclusion of personal values and preferences for the decision of the best therapeutic option [8,45].

The health administration should promote the application of SDM in normal clinical practice, but it is a slow and challenging process [17,18,46,47,48]. It requires developing robust, valid and reliable methodological tools, specific training of professionals, and providing the time and environment to be put into practice [4,8,48,49,50]. The perceived lack of time as a barrier for SDM is not an issue when the consultations are conducted in a structured way towards SDM, and the physicians are trained to do so [47]. Clinical practice guidelines and consensus would play a fundamental role in guiding physicians in practice it [19]. This study identifies a very positive attitude towards SDM on the part of health professionals, who, aware of the usefulness of SDM, and its impact on the quality of care, insist on the need for training, resources and time to be able to put it into practice, with a marked coincidence between professionals from such different social and health contexts as Argentina and Spain. This study has not investigated Argentina and Spain’s cultural differences, so it would be necessary to carry out another study. However, we could conclude that Argentine Healthcare seems to be more privatised than Spanish, which could influence a more significant presence of time and resources for SDM in Argentine Healthcare.

## 5. Conclusions

The professionals involved in treating BC had a high level of knowledge and a positive attitude towards SDM. Its reported application was greater in Spain than in Argentina and in breast units. Lack of time was identified as the main obstacle to its implementation. Health administrations should provide the necessary training and material and human resources for the effective implementation of SDM in the BC care.

## Figures and Tables

**Table 1:** Description of the participants stratified according to their nationality.

	Argentina	Spain	Total	*p*-Value
**Gender**				
Men	121 (51.27%)	97 (44.10%)	218 (47.80%)	*p* = 0.125
Women	115 (48.73%)	123 (55.90%)	238 (52.19%)	
**Total**	236 (100%)	220 (100%)	456 (100%)	
**Age**				
<35 yo	130 (54.62%)	80 (36.36%)	210 (45.85%)	*p* = 0.001
35–50 yo	66 (27.73%)	105 (47.73%)	171 (37.35%)	
51–65 yo	16 (6.72%)	17 (7.73%)	33 (7.20%)	
>65 yo	26 (10.93%)	18 (8.18%)	44 (9.60%)	
**Total**	238 (100%)	220 (100%)	458 (100%)	
**Professional career period**				
MR	0 (0%)	8 (3.63%)	8 (1.75%)	*p* = 0.001
MAS	169 (71.00%)	127 (57.73%)	296 (64.63%)	
Head of Service	67 (28.99%)	74 (33.64%)	141 (30.78%)	
Other	2 (0.01%)	11 (5%)	13 (2.84%)	
**Total**	238 (100%)	220 (100%)	458 (100%)	
**Speciality**				
General Surgery	0 (0%)	126 (56.25%)	126 (27.27%)	*p* = 0.001
Plastic Surgery	72 (30.25%)	61 (27.23%)	133 (28.78%)	
Mastology *	122 (51.26%)	0 (0%)	122 (26.41%)	
Others Speciality	44 (18.49%)	37 (16.52%)	81 (17.54%)	
**Total**	238 (100%)	224 (100%)	462 (100%)	
**Kind of service**				
Breast Unit	131 (39.70%)	199 (88.83%)	330 (71.42%)	*p* = 0.001
Without Breast Unit	107 (81.06%)	25 (11.16%)	132 (28.57%)	
**Total**	236 (100%)	224 (100%)	462 (100%)	
**Hospital**				
Public	94 (39.50%)	172 (76.79%)	266 (57.58%)	*p* = 0.001
Private	144 (60.50%)	52 (23.21%)	196 (42.42%)	
**Total**	238 (100%)	224 (100%)	462 (100%)	
**BC cases/year/hospital**				
<100	106 (44.54%)	54 (24.66%)	160 (35.01%)	*p* = 0.001
100–149	52 (21.85%)	41 (18.72%)	93 (20.35%)	
150–199	30 (12.61%)	32 (14.61%)	62 (13.56%)	
200–249	19 (7.98%)	24 (10.96%)	43 (9.40%)	
>250	31 (13.02%)	68 (31.05%)	99 (21.66%)	
**Total**	238 (100%)	219 (100%)	457 (100%)	
**BC cases/year/doctor**				
<100	151 (63.44%)	94 (41.96%)	245 (53.03%)	*p* = 0.001
100–149	42 (17.65%)	48 (21.42%)	90 (19.48%)	
150–199	15 (6.30%)	13 (5.80%)	28 (6.06%)	
200–249	12 (5.05%)	14 (6.25%)	26 (5.63%)	
>250	18 (7.56%)	38 (16.96%)	56 (12.12%)	
NSNC	0 (0%)	17 (7.58%)	17 (3.68%)	
**Total**	238 (100%)	224 (100%)	462 (100%)	
**% of use of the SDM**				
<33%	49 (20.85%)	19 (8.72%)	68 (15.01%)	*p* = 0.001
33–66%	53 (22.55%)	28 (12.84%)	81 (17.88%)	
>66%	67 (28.51%)	149 (68.35%)	216 (47.69%)	
N/A	66 (28.09%)	22 (10.09%)	88 (19.42%)	
**Total**	235 (100%)	218 (100%)	453 (100%)	

* Speciality only recognized in Argentina. Abbreviations: BC (Breast Cancer), MAS (Medical Area Specialist), MR (Medical Resident), N/A (no answer), SDM (shared decision-making), yo (years old).

**Table 2:** Average response values for each survey question.

	Survey Questions	Mean (CI 95%)	Argentina	Spain	*p*-Value
1	I am familiar with the concept and rationale of Shared Decision Making (SDM)	4.43 (4.36–4.50)	4.51 (4.42–4.60)	4.33 (4.22–4.45)	*p* = 0.027
2	The SDM is a necessary survey to provide quality assistance.	4.48 (4.42–4.55)	4.45 (4.36–4.54)	4.51 (4.42–4.61)	*p* = 0.289
3	The importance of SDM increases when there are several treatment options with similar outcomes, where the selection of one or another option depends on the patient’s preferences.	4.44 (4.37–4.50)	4.43 (4.34–4.52)	4.44 (4.35–4.54)	*p* = 0.741
4	All physicians should ask their patients exactly how they would like to participate in decision-making.	4.29 (4.22–4.36)	4.32 (4.22–4.41)	4.26 (4.16–4.36)	*p* = 0.429
5	SDM increases patient satisfaction, improves cost-effectiveness and reduces malpractice claims.	4.35 (4.28–4.41)	4.34 (4.25–4.27)	4.36 (4.23–4.44)	*p* = 0.708
6	SDM is a basic element in the physician’s relationship with breast cancer (BC) patients.	4.58 (4.51–4.64)	4.79 (4.72–4.85)	4.33 (4.23–4.44)	*p* = 0.001
7	All doctors should inform their patients about the different treatment options available for their health problem.	4.61 (4.55–4.67)	4.57 (4.48–4.67)	4.66 (4.58–4.73)	*p* = 0.211
8	All doctors should explain all treatment options to their patients, including the possibility of not providing any treatment at all.	4.62 (4.56–4.69)	4.79 (4.71–4.84)	4.44 (4.32–4.55)	*p* = 0.001
9	All doctors should explain to their patients the benefits, risks and side effects of possible treatments.	4.72 (4.67–4.78)	4.77 (4.71–4.83)	4.67 (4.58–4.75)	*p* = 0.036
10	All doctors should help their patients understand all the information provided to them.	4.52 (4.46–4.59)	4.35 (4.25–4.44)	4.73 (4.66–4.80)	*p* = 0.001
11	All doctors should ask their patients which treatment option they prefer.	4.32 (4.25–4.38)	4.19 (4.11–4.27)	4.46 (4.37–4.55)	*p* = 0.001
12	Most patients feel that the doctor is the best person to decide on the best treatment option.	4.38 (4.31–4.44)	4.57 (4.49–4.65)	4.15 (4.07–4.24)	*p* = 0.001
13	All doctors should give their patients enough time to assess the different treatment options.	4.38 (4.32–4.45)	4.25 (4.14–4.36)	4.54 (4.46–4.62)	*p* = 0.001
14	All doctors should choose the treatment option together with their patients.	4.29 (4.21–4.37)	4.35 (4.24–4.45)	4.22 (4.11–4.34)	*p* = 0.135
15	All doctors should agree with their patients to monitor their process.	3.80 (3.71–3.89)	3.64 (3.53–3.80)	3.98 (3.84–4.11)	*p* = 0.001
16	My Unit has experience in the use of SDM in breast cancer.	3.80 (3.71–3.88)	3.65 (3.54–3.76)	3.97 (3.85–4.09)	*p* = 0.001
17	My Unit has a specific consultation to explain treatment options and facilitate SDM.	3.34 (3.24–3.44)	3.41 (3.29–3.53)	3.26 (3.10–3.42)	*p* = 0.179
18	My Unit has the necessary time to practice the practice of MDS in the care of the BC	3.45 (3.35–3.55)	3.63 (3.50–3.76)	3.24 (3.09–3.40)	*p* = 0.001
19	My Unit has the necessary materials to practice the SDM in the BC	3.46 (3.37–3.55)	3.61 (3.49–3.72)	3.29 (3.15–3.43)	*p* = 0.001
20	My hospital should promote more patient communication and the BC	3.96 (3.88–4.04)	3.98 (3.87–4.08)	3.93 (3.82–4.05)	*p* = 0.799
21	In general, there should be more training on patient communication and BC	4.33 (4.27–4.40)	4.41 (4.33–4.48)	4.25 (4.15–4.35)	*p* = 0.023
22	SDM can be useful for private health care, but it has no application in public health care, the patient cannot decide on the most efficient treatment option.	2.10 (2.00–2.20)	2.49 (2.34–2.64)	1.65 (1.53–1.76)	*p* = 0.001
23	In the future, there will be an increasing application of SDM in BC care.	4.33 (4.27–4.40)	4.34 (4.25–4.42)	4.33 (4.23–4.43)	*p* = 0.910

Abbreviations: CI (confidence interval).

## Data Availability

Not applicable.

## Appendix A

Cuestionario sobre la práctica de la toma de decisiones compartida (TDC) en el tratamiento del cáncer de mama (CM).

**Instrucciones:**

Estimado compañero, estamos analizando el uso de la TDC en el CM. Nuestro objetivo es evaluar los conocimientos y el uso de la TDC en el tratamiento del CM por los profesionales sanitarios, y para ello te pedimos que respondas las siguientes cuestiones. En ningún momento te pediremos ningún dato personal y por supuesto trataremos toda la información de acuerdo con la Ley Orgánica 3/2018, de 5 de diciembre, de Protección de Datos Personales y garantía de los derechos digitales. Asumimos que al contestar el cuestionario das tu permiso para la utilización de la información que proporcionas, y te agradecemos enormemente tu colaboración.

Selecciona la respuesta más acorde.

◯ Sexo:
  ◯ Hombre.
  ◯ Mujer.
◯ Edad:
  ◯ <35 años.
  ◯ 36–50 años.
  ◯ 51–65 años.
  ◯ >65 años.
◯ ¿En qué periodo de su carrera profesional se encuentra?
  ◯ Médico Interno Residente (MIR).
  ◯ Facultativo Especialista de Área (FEA).
  ◯ Responsable de Servicio o Unidad.
  ◯ Otros: __________
◯ ¿Qué tipo de especialidad tiene?
  ◯ Cirugía General
  ◯ Ginecología y Obstetricia
  ◯ Anatomía Patológica
  ◯ Radiología
  ◯ Oncología
  ◯ Medicina de Familia
  ◯ Otros: _________________
◯ Tipo de Servicio o Unidad donde desarrolla su ejercicio:
  ◯ Servicio de Cirugía General y del Aparato Digestivo o Ginecología y Obstetricia.
  ◯ Servicio de Cirugía General o Ginecología con especial dedicación a la Mama.
  ◯ Unidad de Mama.
  ◯ Otros: __________________
◯ Ámbito donde desarrolla su ejercicio (puede marcar más de una opción):
  ◯ Hospital público o perteneciente al Servicio Sanitario Público.
  ◯ Compañía u Hospital Privado.
  ◯ Otras situaciones: ______________________

Si señalaste la primera opción, Hospital Público, puedes indicar a que categoría corresponde:

◯ Hospital regional o de referencia
◯ Hospital de especialidades
◯ Hospital de área o comarcal
◯ Hospital de alta resolución

◯ Número de casos de Cáncer de Mama atendidos por su Servicio o Unidad al año:
  ◯ <100
  ◯ 100–149
  ◯ 150–199.
  ◯ 200–249
  ◯ 250 o más
◯ Número de pacientes con Cáncer de Mama atendidos en consulta por usted al año:
  ◯ <100
  ◯ 100–149
  ◯ 150–199.
  ◯ 200–249
  ◯ 250 o más
◯ Porcentaje de casos de Cáncer de Mama atendidos en su hospital en los que se realiza una toma de decisiones compartidas
  ◯ <33%
  ◯ 33–66%
  ◯ >66%
  ◯ No lo sé

Seleccione la respuesta más acorde con su opinión o experiencia. Intente no dejar preguntas en blanco:

		**Totalmente en Desacuerdo**	**En Desacuerdo**	**Ni de Acuerdo** **ni en Desacuerdo**	**De Acuerdo**	**Totalmente de Acuerdo**
1	Conozco el concepto y los fundamentos de la Toma de Decisiones Compartida (TDC)	1	2	3	4	5
2	La TDC es una herramienta necesaria para proporcionar una asistencia de calidad.	1	2	3	4	5
3	La importancia de la TDC aumenta cuando existen diversas opciones de tratamiento con resultados similares, en las que la selección de una u otra opción depende de las preferencias del paciente.	1	2	3	4	5
4	La TDC aumenta la satisfacción del paciente, mejora la rentabilidad y reduce las demandas por negligencia.	1	2	3	4	5
5	La TDC es un elemento básico en la relación del cirujano con los pacientes con Cáncer de Mama (CM).	1	2	3	4	5
6	Todos los médicos deberían preguntar a sus pacientes exactamente cómo les gustaría participar en la toma de decisiones.	1	2	3	4	5
7	Todos los médicos deberían informar a sus pacientes sobre las diferentes opciones de tratamiento existentes para su problema de salud.	1	2	3	4	5
8	Todos los médicos deberían explicar a sus pacientes todas las opciones de tratamiento, incluyendo la posibilidad de no realizar ningún tratamiento.	1	2	3	4	5
9	Todos los médicos deberían explicar a sus pacientes los beneficios, riesgos y efectos secundarios de los posibles tratamientos.	1	2	3	4	5
10	Todos los médicos deberían ayudar a sus pacientes a entender toda la información que se les proporciona.	1	2	3	4	5
11	Todos los médicos deberían preguntar a sus pacientes qué opción de tratamiento prefieren.	1	2	3	4	5
12	La mayor parte de los pacientes considera que el médico es la persona más adecuada para decidir cuál es la mejor opción terapéutica.	1	2	3	4	5
13	Todos los médicos deberían proporcionar a sus pacientes el tiempo suficiente para que puedan valorar las diferentes opciones de tratamiento.	1	2	3	4	5
14	Todos los médicos deberían escoger conjuntamente con sus pacientes la opción de tratamiento.	1	2	3	4	5
15	Todos los médicos deberían consensuar con sus pacientes el seguimiento de su proceso.	1	2	3	4	5
16	Mi Unidad tiene experiencia en el uso de la TDC en cáncer de mama.	1	2	3	4	5
		**Totalmente en Desacuerdo**	**En Desacuerdo**	**Ni de Acuerdo** **ni en Desacuerdo**	**De Acuerdo**	**Totalmente de Acuerdo**
17	Mi Unidad dispone de una consulta específica para explicar las opciones de tratamiento y facilitar la TDC.	1	2	3	4	5
18	Mi Unidad dispone del tiempo necesario para practicar la TDC en la asistencia del CM	1	2	3	4	5
19	Mi Unidad dispone de los materiales necesarios para practicar la TDC en el CM	1	2	3	4	5
20	Mi hospital debería promocionar más la comunicación con el paciente y la TDC	1	2	3	4	5
21	En general, debería haber más formación sobre comunicación con el paciente y la TDC	1	2	3	4	5
22	La TDC puede ser útil para la asistencia sanitaria de carácter privado, pero no tiene aplicación en la asistencia sanitaria pública, el paciente no puede decidir sobre la opción de tratamiento más eficiente	1	2	3	4	5
23	En el futuro se aplicará cada vez más la TDC en la atención al CM	1	2	3	4	5
